# Conflict and tuberculosis in Sudan: a 10-year review of the National Tuberculosis Programme, 2004-2014

**DOI:** 10.1186/s13031-018-0154-0

**Published:** 2018-05-16

**Authors:** Sara A. Hassanain, Jeffrey K. Edwards, Emilie Venables, Engy Ali, Khadiga Adam, Hafiz Hussien, Asma Elsony

**Affiliations:** 1 The Epidemiological Laboratory, House 34, Street 53, New Extension, P.O.BOX 13595, Khartoum, Sudan; 2 Médecins Sans Frontières, Luxembourg Operational Research Unit (LuxOR), Luxembourg City, Luxembourg; 3 Johns Hopkins University, School of Public Health, Baltimore, MD USA; 4 Division of Social and Behavioural Sciences, School of Public Health and Family Medicine, University of Cape Town, Cape Town, South Africa; 5 Directorate General of Planning and International Health - Health Information Federal Ministry of Health-Sudan, Khartoum, Sudan

**Keywords:** Sudan, Conflict, Non-conflict, Tuberculosis programme, Performance, Darfur, Outcomes, Operational research

## Abstract

**Background:**

Sudan is a fragile developing country, with a low expenditure on health. It has been subjected to ongoing conflicts ever since 1956, with the Darfur crisis peaking in 2004. The conflict, in combination with the weak infrastructure, can lead to poor access to healthcare. Hence, this can cause an increased risk of infection, greater morbidity and mortality from tuberculosis (TB), especially amongst the poor, displaced and refugee populations. This study will be the first to describe TB case notifications, characteristics and outcomes over a ten-year period in Darfur in comparison with the non-conflict Eastern zones within Sudan.

**Methods:**

A cross-sectional review of the National Tuberculosis Programme (NTP) data from 2004 to 2014 comparing the Darfur conflict zone with the non-conflict eastern zone.

**Results:**

New case notifications were 52% lower in the conflict zone (21,131) compared to the non-conflict zone (43,826). Smear-positive pulmonary TB (PTB) in the conflict zone constituted 63% of all notified cases, compared to the non-conflict zone of 32% (*p* < 0.001). Extrapulmonary TB (EPTB) predominated the TB notified cases in the non-conflict zone, comprising 35% of the new cases versus 9% in the conflict zone (*p* < 0.001). The loss to follow up (LTFU) was high in both zones (7% conflict vs 10% non-conflict, *p* < 0.001) with a higher rate among re-treatment cases (12%) in the conflict zone. Average treatment success rates of smear-positive pulmonary TB (PTB), over ten years, were low (65-66%) in both zones. TB mortality among re-treatment cases was higher in the conflict zone (8%) compared to the non-conflict zone (6%) (*p* < 0.001).

**Conclusion:**

A low TB case notification was found in the conflict zone from 2004 to 2014. High loss to follow up and falling treatment success rates were found in both conflict and non-conflict zones, which represents a significant public health risk. Further analysis of the TB response and surveillance system in both zones is needed to confirm the factors associated with the poor outcomes. Using context-sensitive measures and simplified pathways with an emphasis on displaced persons may increase access and case notification in conflict zones, which can help avoid a loss to follow up in both zones.

## Background

Conflicts impact health infrastructure and human resources, which can hinder disease prevention and control measures. This escalates the burden of communicable diseases, such as tuberculosis (TB) [1, 2]. In addition, conflicts cause the displacement of populations and impair access to healthcare. This can increase TB transmission, worsen patient outcomes and lead to increasing rates of drug resistance [3, 4]. A study conducted in Nepal found that the poor uptake of TB services was due to war-associated factors [1]. Furthermore, high treatment initiation delays and self-treatment have also been observed in the Somali conflict region of Ethiopia [5]. Displaced populations and refugee settlements are frequently exposed to TB risk factors, which can lead to increased transmission. An example is in Lebanon, where a 27% rise in TB incidence was attributed to the increase in the Syrian refugee population [6]. Likewise, a study in El Salvador reported the incidence rate of TB among internally displaced persons (IDPs) to be three times higher than the reported rate across the rest of the country [7]. Furthermore, refugees and IDPs are at a higher risk of acquiring multi-drug resistant (MDR) TB due to treatment interruption [8, 9]. TB associated mortality has been reported to be higher in conflict zones, as demonstrated by a study from Guinea Bissau that found TB mortality to be three times higher among conflict cohorts in comparison to non-conflict cohorts [10]. A study from Kenya has shown that TB caused 20% more deaths within children aged 5 years and younger within the internally displaced population, in comparison with the children of regular residents [11]. TB was attributed to the deaths of approximately 26% of adult IDPs in Somalia and 38% to 50% of all deaths among adult refugees in refugee camps in eastern Sudan [4].

Sudan is an extremely turbulent country that has suffered from years of civil conflicts, including the North-South and Darfur crises [12]. Sudan has a low health expenditure (6.2% of GDP) and has an estimated TB incident rate of 117 per 100,000 individuals [13]. Notified TB cases have remained relatively constant over the past decade, within the range of 19,817 to 22,097. Moreover, Sudan has a low human immunodeficiency virus (HIV) prevalence, which was reported to be 0.3% as of 2015. However, the prevalence of MDR TB, as reported by Sudan’s National TB programme (NTP) in 2012, was 19% in re-treatment cases and 1.8% in new cases [13]. Another study in north-eastern Sudan reported an MDR prevalence of 6% among new TB cases [14].

The Darfur conflict officially started in 2003, but clashes flared with a greater intensity in 2004, leading to a humanitarian crisis. There have been approximately 1.9 million IDPs since the beginning of the conflict. The highest levels of violence were experienced in 2014 when more than 430,000 people were newly displaced. There have also been reports of deprived health situations, prevalent TB risk factors, low primary health care (PHC) coverage, inadequate health system accessibility and depleted human resources within the Darfur region [15]. Data associated with TB in Darfur are limited, and there is an ever-increasing need to address this within the vulnerable conflict-ridden population. To our knowledge, there are no studies analyzing TB data, over a 10-year period, at a national level within Sudan. In particular, there has not been a comparison between a conflict zone and a non-conflict zone within Sudan. Our hypothesis is that conflicts negatively affect TB prevention and control measures. This study aims to compare overall TB case notifications, treatment outcomes and trends between the conflict region of Darfur and the non-conflict region of eastern Sudan from 2004 to 2014, to determine the TB control status in the conflict zone.

## Methods

### Study design

This is a descriptive cross-sectional review of Sudan’s NTP surveillance aggregated data from 2004 to 2014, within a conflict zone (Darfur) and a non-conflict zone (Eastern Sudan).

### General setting

Sudan is a Sub-Saharan African country with an estimated population of 40 million. It is ranked at 167 out of 188 countries in the Human Development Index (HDI) and has 4.8 million people needing humanitarian assistance because of conflict, environmental and food insecurity factors [16].

### Specific setting

The Darfur conflict zone is a border region that covers an area of 493,180 km^2^ in western Sudan. It has an estimated population of six million people, including five states (North, West, South, East and Central Darfur). The conflict has caused an internal displacement of people, of which at least 60% are children living in camps. It has led to Darfur having approximately 140,000 refugees [17].

The non-conflict zone is a border region in eastern Sudan that covers an area of 324,773 km^2^. It includes three states (Kassala, Gadarif and the Red Sea) with a combined population of approximately 5 million and has not experienced any conflict during 2004-2014. There are over 135,000 refugees and asylum seekers residing in camps [17].

Two zones were chosen for this study to examine the hypothesis that conflicts have a negative impact on TB indicators. The zones were selected because of their similarities, which are as follows:

- A mixed population of settled peasant and nomadic herders
- Prevalent poverty, famine and inequalities at social and political levels, including a lack of access to adequate quality health services
- Widespread inter- and cross-border mobility and displacement, which increase the risk of communicable diseases
- Targeted humanitarian response for vulnerable IDPs and refugees in partnership with local authorities, State Ministries, United Nations (UN) agencies, international and national Non-Governmental Organizations (NGOs)
- Severely compromised health systems
- TB management and surveillance systems functioning similarly across both zones

### TB management and control

TB prevention and control activities are coordinated by the NTP in both the conflict and non-conflict zones. The NTP is managed by a central unit located in Khartoum, with sentinel coordination in each state. Expansion has been achieved through the decentralisation of diagnosis and treatment into TB management units (TBMUs). The services have been integrated into the existing healthcare system, which includes recording, reporting, training, supervision and health education. This allows coverage of an average of 100,000 people per TBMU.

TB laboratory activities are performed in the regular laboratory network composed of the national reference laboratory in the capital Khartoum and state quality control laboratories. They provide cultures and drug susceptibility. TB patients are treated free of charge and in accordance with the World Health Organization’s (WHO) recommended Directly Observed Treatment, Short Course TB control strategy (DOTS). TB services are provided through health services in hospitals, health centres, assigned NGOs and health insurance centres [18]. In the Darfur conflict zone, approximately 81% of TBMUs are governmental, 9% are run by military troops, 8% by NGOs and the remaining are insurance centres. In the Eastern zone, approximately 85% of TBMUs are governmental, 9% are NGOs, 4% are military centres, and 2% are insurance centres. NGOs are crucial in strengthening the TB community systems in both zones by tracing patients lost to follow up, conducting contact tracing and active case finding. Moreover, they also handle referrals with the help of trained community health volunteers.

The surveillance system is standard across Sudan, including the conflict-associated zones. Data collection occurs at TBMU’s, which utilise patient cards and registers, with the reporting of aggregated data to state and central authorities occurring quarterly. This reporting gathers information regarding the case, age, gender and outcomes. MDR TB care is provided in the Abu-Anga central hospital in the state of Khartoum. In 2013-2014, a change of NTP management was introduced with oversight provided by the Communicable and Non-Communicable Disease Department (CNDC).

### Study population

The study included all centrally registered TB patients from the two study zones, which are Darfur and the Eastern states, between 2004 and 2014.

### Data collection and analysis

The data were validated at various points, including the point of entry, at the central NTP level, and through inspections of registries for missing data and consistency. Quarterly aggregated data (number of cases per TB type, age and gender) were reviewed by year and state for conflict and non-conflict zones, covering the period of 2004 to 2014. The data were processed before the analysis to validate the consistency between the total number of TB cases (new and re-treated), as well as cohort registered treatment outcomes. The variables that were collected include the number of registered cases, the epidemiological characteristics of registered cases (gender and age), the type and category of TB, and treatment outcomes. Standardised definitions for these variables were consistent with the International Union Against Tuberculosis and Lung Disease (The Union) and the WHO.

### Statistical analysis

The data were extracted from the NTP reporting platforms into Microsoft Excel (Microsoft Corp, Redmond, Washington, USA), computerised and entered in STATA version 13.0 (Stata Corp, College Station, Texas, USA). Using descriptive frequency analysis, the demographic and clinical characteristics, and treatment outcomes were analysed in relation to the conflict and non-conflict zones. The data were cross-tabulated using the chi-square test to examine relationships. A T-test was applied and a *p*-value lower than 0.05 was considered significant. The associated trends comparing conflict and non-conflict zones were calculated.

## Results

### Case notification

Between 2004 and 2014 a total of 21,131 new TB cases were reported in the conflict zone (Darfur) versus 43,826 in the non-conflict zone. There were 1822 notified TB re-treatment cases from the conflict and 2024 from the non-conflict zone, as shown in Table 1.

**Table 1:** Case notification classification and treatment outcomes of new and re-treatment tuberculosis, TB, cases in conflict and non-conflict zones in Sudan, 2004-2014

Clinical characteristics (New cases)	Conflict zone *N* = 21,131 (100%)	Non-conflict *N* = 43,826 (100%)	*p-*value
PTB smear positive	13,283 (63)	14,016 (32)	< 0.001
PTB smear negative	5309 (25)	12,680 (30)	< 0.001
EPTB	1963 (9)	15,378 (35)	< 0.001
PTB (not done)	576 (3)	1392 (3)	0.073
Treatment outcomes (New cases)	Conflict *N* = 20,326 n (%)	Non-conflict *N* = 43,826 n (%)	
Treatment success^a^	13,498 (66)	28,645 (65)	< 0.001
Died	422 (2)	1124 (3)	< 0.001
Treatment failure	139 (1)	156 (< 1)	0.703
LTFU	1379 (7)	4709 (10)	< 0.001
Transferred out	592 (3)	2141 (5)	0.001
Not evaluated	4296 (21)	7051 (16)	0.526
Clinical characteristics (Re-treatment cases)	Conflict zone *N* = 1822 (100%)	Non-conflict *N* = 2024 (100%)	
After Relapse	912 (50)	1412 (70)	0.005
After LTFU^a^	669 (37)	314 (16)	0.014
After Failure	155 (8)	148 (7)	0.804
Others^b^	86 (5)	150 (7)	0.276
Treatment outcomes (Re-treatment cases)	Conflict zone *N* = 1822 n (%)	Non-conflict *N* = 2024 n (%)	
Treatment success^a^	693 (38)	911 (45)	< 0.001
Died	146 (8)	121 (6)	< 0.001
Treatment failure	163 (9)	122 (6)	< 0.001
LTFU	218 (12)	202 (10)	< 0.001
Transferred out	200 (11)	243 (12)	0.986
Not evaluated	402 (22)	425 (21)	0.713

^a^*PTB* Pulmonary TB, *EPTB* Extrapulmonary TB, *LTFU* Loss to follow up, Treatment success = Cured and treatment completed cases

^b^all cases that are registered whom their definition is not consistent with other re-treatment definitions

**Fig. 1:**
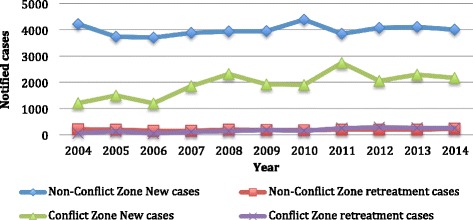
New and retreatment case notification in conflict and non-conflict zones in Sudan, 2004-2014

As shown in Fig. 1, the non-conflict zone surpassed the conflict zone in the number of notified new cases per year with a range of 4387-3698 versus 2734-1196, between 2004 and 2014. The average notification rates of new cases during this time were 88 and 37 per 100,000 population in the non-conflict and conflict zones respectively. Furthermore, the average notification rates of re-treatment cases between 2004 and 2014 were 4.5 and 3 per 100,000 population in the non-conflict and conflict zones respectively.

### Demographic characteristics

Of the reported new cases, 10% were in children aged 15 years and younger in the conflict zone, compared to 5% of cases in the non-conflict zone. Age was unrecorded for 18% and 22% of new TB cases in the conflict and non-conflict zones respectively. Gender was not recorded for almost 29% and 30% of the new TB cases in the conflict and non-conflict zones respectively. Within the new TB cases there were 6891 (59%) males and 4844 (41%) females in the conflict zone, compared to 7921 (62%) males and 4875 (38%) females in the non-conflict zone (*p* < 0.001). The data were not disaggregated by age or gender for the re-treatment TB cases in either zone.

### Clinical characteristics

#### TB type and category

The distribution of tuberculosis types as proportions among newly notified cases (Table 2) shows that the new cases with reported diagnosis by TB type and category (21,131 conflict, 43,826 non-conflict) were more smear positive pulmonary TB in the conflict zone (63%) compared to the non-conflict zone (32%, *p* < 0.001).

**Table 2:** Distribution of tuberculosis types as proportions among newly notified cases in conflict and non-conflict zones, Sudan 2004-2014

	Smear-positive*		Smear-negative*		Extrapulmonary*	
Year	Conflict 13,283 (100%)	Non-conflict 14,016 (100%)	Conflict 5309 (100%)	Non-conflict 12,680 (100%)	Conflict 1963 (100%)	Non-conflict 15,738 (100%)
2004	992 (82%)	1497 (36%)	102 (8%)	1456 (35%)	111 (19%)	1262 (30%)
2005	958 (64%)	1461 (39%)	307 (21%)	1198 (32%)	226 (15%)	1077 (29%)
2006	764 (64%)	1338 (36%)	312 (26%)	1302 (35%)	120 (10%)	1058 (29%)
2007	1218 (66%)	1385 (36%)	474 (26%)	1240 (32%)	164 (9%)	1261 (32%)
2008	1740 (75%)	1393 (35%)	365 (16%)	1099 (28%)	198 (9%)	1351 (34%)
2009	1364 (71%)	1292 (33%)	375 (19%)	992 (25%)	157 (8%)	1517 (38%)
2010	1462 (77%)	1259 (29%)	202 (11%)	1218 (28%)	206 (11%)	1731 (39%)
2011	1690 (62%)	1132 (29%)	690 (25%)	831 (22%)	198 (7%)	1652 (43%)
2012	1085 (53%)	1227 (30%)	693 (34%)	932 (23%)	194 (9%)	1678 (41%)
2013	980 (43%)	1066 (26%)	969 (42%)	1083 (26%)	196 (9%)	1695 (41%)
2014	1030 (47%)	966 (24%)	820 (38%)	1329 (33%)	193 (9%)	1456 (36%)

*TB patterns were reported as proportions from the total number of notified new cases each year in the conflict zone and non-conflict zone during 2004-2014

EPTB was the most common type reported in the non-conflict zone comprising 35% of the new cases, as shown in Table 2. There was a significant difference in the overall TB smear negative proportions in the conflict zone (25%) versus the non-conflict zone (30%, *p* < 0.001), as shown in Table 1.

Re-treatment cases constituted 7% and 4% of all notified cases in the conflict and non-conflict zones respectively. Among re-treatment cases, 37% were classified as retained after the LTFU in the conflict zone compared to 16% in the non–conflict zone (*p* = 0.014), as seen in Table 1.

#### Treatment outcomes

**Fig. 2:**
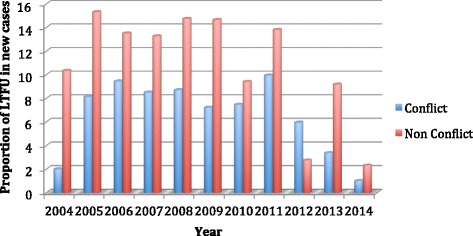
Lost to follow up rates among new cases in conflict and non-conflict zones Sudan 20,014-2014: * Lost to follow up rates (LTFU) were calculated as the total of LTFU/number of registered cases of treatment outcomes * 100 per year in two zones over 2004-2014

**Fig. 3:**
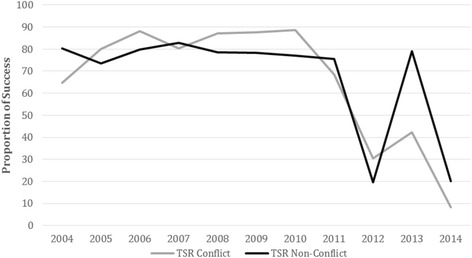
Treatment success rates for pulmonary smear positive tuberculosis in conflict and non-conflict zones of Sudan, 2004-2014. *Treatment success rates were calculated as totals of cured and completed treatment cases/ number of registered cases of treatment outcomes * 100 per year in two zones over 2004-2014

The LTFU rates among new cases were 7% and 10% for the conflict and non-conflict zones, respectively (*p* = < 0.001), as shown in Fig. 2. Reported failures among re-treatment cases were 9% and 6% between 2004 and 2014 for the conflict and non-conflict zones respectively (*p* < 0.001). Treatment success rates (TSR) of new cases averaged 66% in the conflict zone and 65% in the non-conflict zone between 2004 and 2014 (*p* < 0.001), as shown in Fig. 3. A steady decrease in TSR of all smear-positive PTB cases was observed from 2010 in both the conflict and non-conflict zones.

## Discussion

To the best of our knowledge, this is the first study describing the notification, characteristics and outcomes of TB cases, between the Darfur conflict region and the Eastern non-conflict zones in Sudan over a ten-year period. Some of the findings are consistent with the hypothesis that conflicts are likely to have a negative impact on TB response measures. However, TB control indicators, including LTFU and treatment success among new TB cases, were worse in the non-conflict zone. Protracted conflicts, ongoing humanitarian crises and associated low expenditure on health are likely to have exacerbated an already unfavourable condition for TB control indicators and disease outcomes.

### Challenges for TB control within the conflict zone

The study identified a lower number of cases and notification rates of new and re-treatment TB in the Darfur conflict zone. This may reflect a vast and inaccessible community within the conflict zone and a considerable gap in the access to healthcare. Poverty and high direct non-medical costs for the patient can lower accessibility to care in both conflict and non-conflict zones [19]. Nevertheless, turbulence and insecurity in the conflict zone may have further negative effects on the access to healthcare and further exclude communities from timely TB management. Specifications on socio-economic status, residency status, and IDP and refugee camps were not differentiated at the surveillance level. Therefore, no identification regarding these access-associated factors could be obtained for those displaced in the Darfur conflict zone.

There was a very high proportion of smear-positive PTB, comprising 63% of all newly notified cases in the conflict zone. This proportion is higher than the 56% positive results reported among 1797 registered patients in the seven states of Sudan between 1998 and 2000 [20]. Here, the crucial finding is that the there is a higher proportion of smear-negative and EPTB cases in the non-conflict zone compared with the conflict zone. As there is a reported healthcare gap in Darfur, the low number of reported EPTB cases in the conflict zones compared to the non-conflict zones could be attributed to the lack of capacity in the disrupted zone to execute adjunctive EPTB diagnosis [15]. This finding needs to be further analysed with regards to the TB associated symptoms and diagnostic criteria measures because this TB pool could be expanded by conflict associated delay in seeking care [5, 21].

The identification of all TB disease types in conflict settings can be improved by strengthening the capacity of frontline care in tackling presumptive TB and implementing simple and rapid diagnostics. The proportion of re-treatment cases was 7% of all notified cases in the conflict zone, which is less than the 16.2% and 9.1% reported among the IDPs and the settled population in Khartoum State [22]. There are higher rates of treatment failure and LTFU among the re-treatment cases in the Darfur conflict zone compared to the non-conflict zone. This can increase the risk of drug resistance, which is particularly serious for displaced populations who are already exposed to risk factors associated with a poor socio-economic context, which include the risk of higher HIV prevalence while crossing to neighbouring countries [23].

Children make up an important demographic in TB epidemiology, especially those living in poor socio-economic conditions with low diagnostic capacities [16]. Children constituted 5% and 10% of notified cases in the non-conflict and conflict zones respectively. This may be due to the lower cases notified in general within the conflict zone. These figures are less than the 13% reported in Ethiopia but within the expected range of 5-15% in low-income countries [24]. The Surveillance data are not fully disaggregated by age, especially among re-treatment cases, highlighting a limitation of the TB programme in addressing childhood TB.

### Challenges for TB control within the conflict and non-conflict zones

We observed high LTFU rates among reported new and re-treatment cases throughout 2004-2014 in both zones (7% and 10% in the conflict zone compared to 12% and 10% in the non-conflict zone). High LTFU rates can lead to increased disease transmission, greater rates of smear positivity, drug resistance, chronic pulmonary impairment and increased mortality rates [25]. These findings are supported by the study from Galkayo, which found a correlation between the 10% LTFU rate in Somalia and weak health system management [3].

In Botswana, EPTB was found to be significantly associated with a higher LTFU, which may explain the high proportion of EPTB cases of 36% in the newly reported cases in the non-conflict zone [26]. A study in Ethiopia has identified living in a rural setting as an independent predicator for LTFU [27]. The rural predominance, and peasant and nomadic populations in both zones indicate the vital need of context-sensitive TB services to reduce the LTFU in both zones.

The TSR of new cases were satisfactory in both zones during 2005-2010, and better rates were observed in the conflict zone. This could be related to the smaller cohort and the higher proportion of smear-positive cases, where the cohort is more likely to be more ailing, and therefore more likely to complete treatment. A comparative study of treatment outcomes in Khartoum State showed significantly higher success rates among the displaced (74%) compared to settled populations (64%) [22].

However, a steady decrease in the TSR of all smear-positive PTB cases was observed in both zones from 2010. This is operationally related to the high proportions of unfavourable outcomes, including LTFU and non-evaluated cases. This deterioration in performance in 2010 and the subsequent decrease in TSR in 2012 could be related to challenges in data management and the lack of monitoring at higher surveillance levels. These pitfalls may hinder sustainable development goals and exacerbate the TB-poverty-vulnerability cycle, especially in conflict settings.

Our findings highlight an urgent need of determined efforts to investigate the associated factors and reasons behind these sub-optimal parameters in both zones. There is a need to revise TB control with “context-sensitive” approaches that simplify pathways to care while improving treatment adherence and tracing strategies. These efforts are needed in both zones to curb the emerging public health threats related to TB.

A limitation of this study is the dependence on routine TB aggregated data, which does not allow for the disaggregation of socioeconomic specifications, displacement, and re-treatment cases by age and gender. Additionally, the lack of data reporting has likely impacted the results to an uncertain degree, because the surveillance does not differentiate between non-functioning and zero notification centres. The strengths of the study include the ten-year timespan of the TB data while assuming a steady population, and surveillance system within both zones.

## Conclusions

There were lower case notification rates and lower proportions of EPTB and smear-negative cases in the conflict zone. However, the TB control indicators, including LTFU and treatment success among new TB cases were found to be worse in the non-conflict zone. Further study is needed to identify determinant factors and causal relations behind these findings, along with context-tailored strategies that increase case finding and promote improved treatment outcomes in both conflict and non-conflict zones. Potential opportunities include the introduction of rapid diagnostic testing and improved frontline healthcare. Local communities could strengthen TB case finding, treatment adherence and contact tracing to avoid LTFU and improve treatment outcomes. Lastly, the inclusion of the place of residence and distance specifications in the surveillance system would support the monitoring of TB burden and access, especially among displaced, refugee, and mobile populations.

## Abbreviations

CNDC.: Communicable and Non-Communicable Disease Department
DOTS: Directly observed treatment strategy
EPTB: Extrapulmonary TB
ERB: Ethics Review Board
FMOH: Federal Ministry of Health
IDPs: Internally displaced persons
LTFU: Loss to follow up
MDR: Multi-drug resistant
MSF: Médecins Sans Frontières
NGO: Non-governmental Organization
NTP: National TB Programme
PHC: Primary health care
PTB: Pulmonary TB
SDGs: Sustainable development goals
TB: Tuberculosis
TBMU: TB Management Unit
TSR: Treatment success rate
UN: United Nations
